# Myositis ossificans in the infraspinatus muscle: The key to diagnosis

**DOI:** 10.1002/ccr3.2439

**Published:** 2019-09-27

**Authors:** Eliza Stavride, Antonia Bintoudi, Sofia–Chrysovalantou Zagalioti, Nikiforos Galanis

**Affiliations:** ^1^ Department of Radiology Papageorgiou General Hospital Thessaloniki Greece; ^2^ Division of Sports Medicine Department of Orthopaedics Papageorgiou General Hospital Medical School Aristotle University of Thessaloniki Thessaloniki Greece

**Keywords:** heterotopic ossification, infraspinatus muscle, myositis ossificans, zonal pattern, zone phenomenon

## Abstract

The zone phenomenon is the most important diagnostic feature in differentiating myositis ossificans from malignancies such as osteosarcomas, which calcify from the center to the periphery and its presence in our late‐stage lesion was the key to diagnosis.

## Graphical abstract

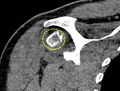

A 53‐year‐old man presented with a 1‐year history of persistent, durable right shoulder pain. No trauma history was admitted. Initial workup consisted of a magnetic resonance imaging (MRI) of the right shoulder. Both T1‐ and T2‐weighted images revealed a round, well‐defined, inhomogeneous mass with signal intensity similar to fat, localized in the infraspinatus muscle. A mild, low‐signal, peripheral rim was evident (Figure 1). The scapular‐Y radiograph demonstrated an extraskeletal ossified area, adjacent to the acromioclavicular joint (Figure 2) and computed tomography scan finally established the diagnosis (Figures 3,4). Myositis ossificans (MO) is a benign, solitary, ossifying soft tissue mass typically occurring within the musculature. Infraspinatus muscle is an extremely rare location of the lesion.^1^

**Figure 1:**
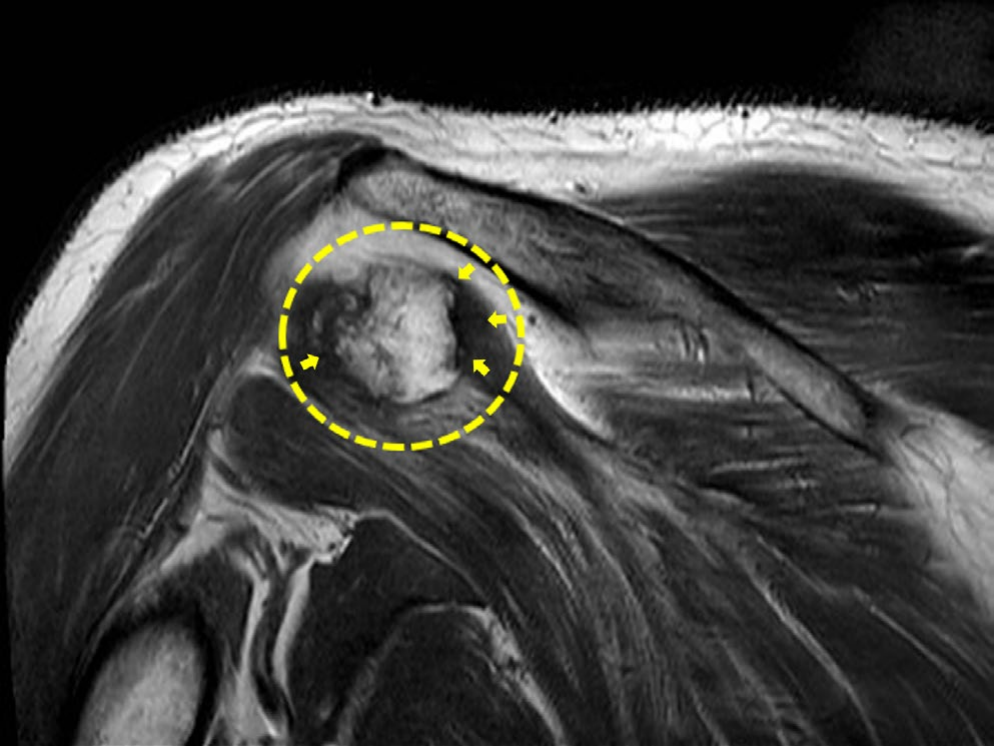
Coronal T1‐weighted MR image demonstrating a round inhomogeneous mass with signal intensity similar to fat, within in the infraspinatus muscle and consistent with a pattern of characteristic mature lamellar bone (yellow circle). A mild, low‐signal, peripheral rim is evident (yellow arrows). No surrounding edema is observed

**Figure 2:**
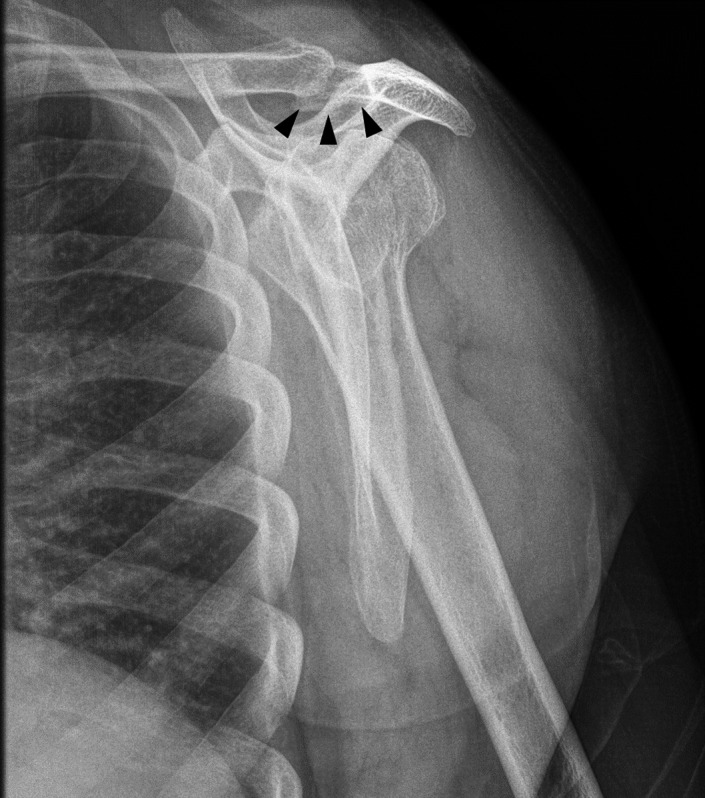
Scapular‐Y radiograph where a faint, extraskeletal, ossified area is depicted, underneath the acromioclavicular joint (black arrowheads)

**Figure 3:**
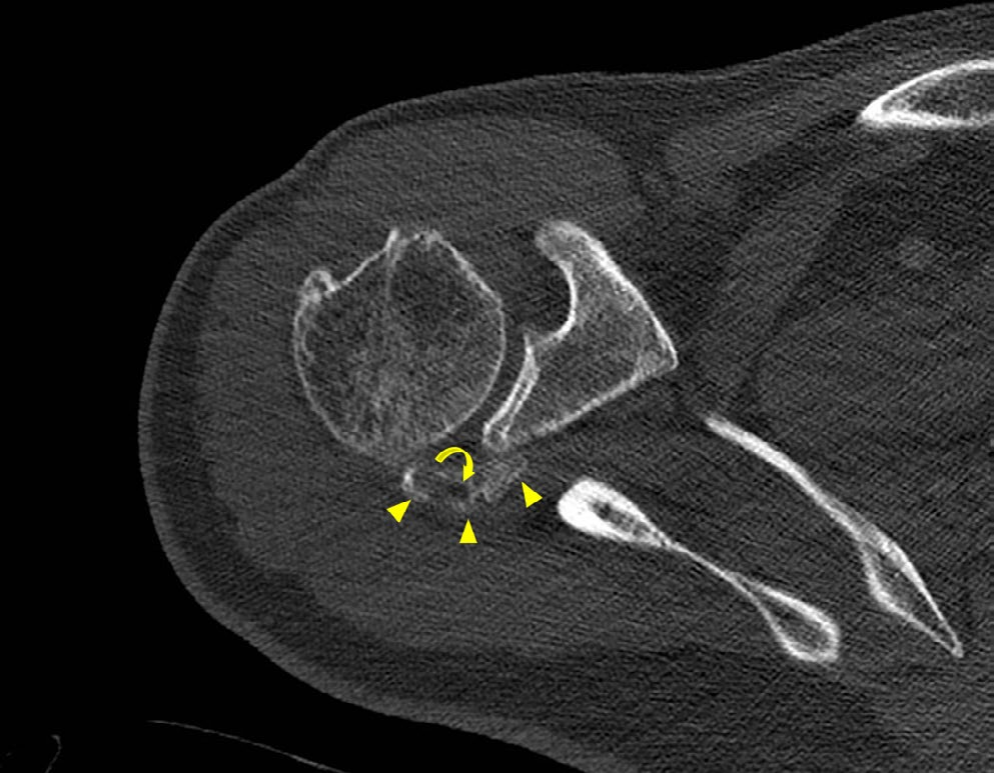
Axial computed tomography image showing an ossified mass localized in the infraspinatus muscle (yellow arrowheads). The center (curved arrow) appears with lower attenuation than the periphery of the lesion due to less mineralization

**Figure 4:**
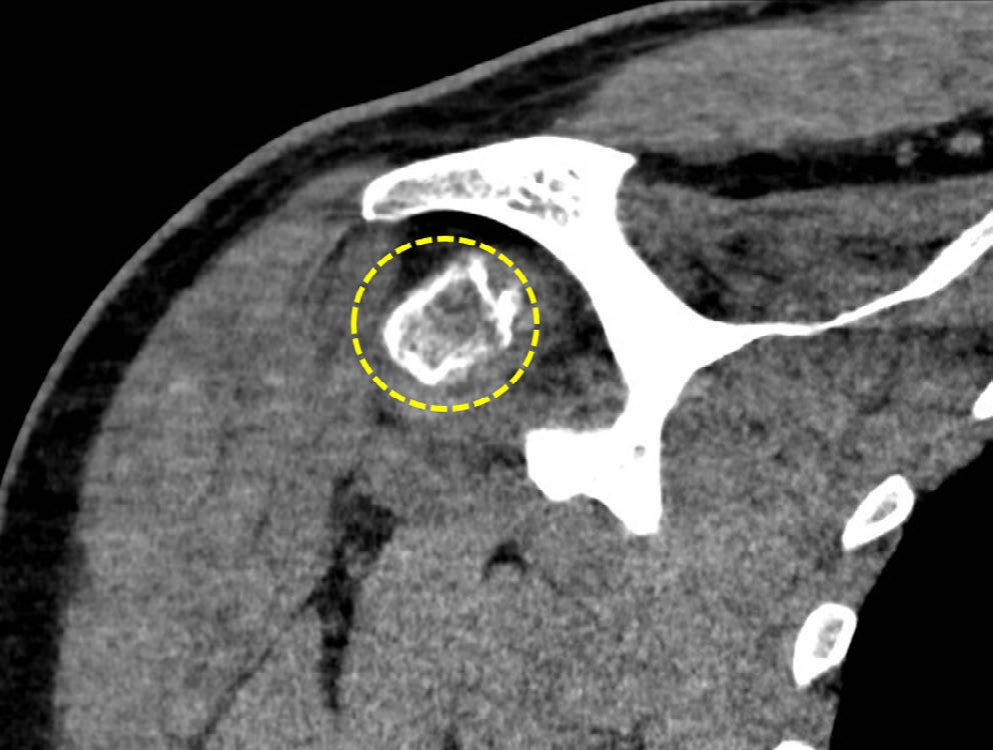
Computed tomography image (soft tissue window) showing the extension of the mass at the coronal reformation (yellow circle). Peripheral mineralization of the lesion is demonstrated. No soft tissue edema is observed

Myositis ossificans typically has three stages of progression: the early stage, the intermediate stage, and the mature stage. Each of these stages is characterized by its own radiographical findings. In early stage, plain radiographs are usually normal or faint calcifications may be seen. During the intermediate stage, a peripheral calcification may be demonstrated whereas a mature lesion may appear with diffuse calcification, as the osteoid becomes mineralized. T1‐weighted images will demonstrate iso‐ to hyperintense‐signal, and T2‐weighted images will demonstrate a hyperintense inhomogeneous mass usually with diffuse surrounding edema. As the lesion progresses, it matures and calcifies from the periphery to the center, the so‐called “zone phenomenon. This centripetal pattern is the most important diagnostic feature in differentiating MO from malignancies such as osteosarcomas, which calcify from the center to the periphery, and its presence in our late‐stage lesion was the key to diagnosis. Additional imaging characteristics to this direction is the lack of invasion of adjacent tissues and the absence of muscle fibers within the lesion, often seen in tumors.^1^, ^2^

## CONFLICT OF INTEREST

None declared.

## AUTHOR CONTRIBUTIONS

Eliza Stavride: reviewed the literature, wrote the manuscript, and edited the images. Antonia Bintoudi: established the diagnosis, made a contribution to drafting, and reviewed the manuscript. Sofia – Chrysovalantou Zagalioti: contributed to review of the literature and drafting. Nikiforos Galanis: contributed to patient care and revised the manuscript.
